# Modeling the structural implications of an alternatively spliced *Exoc3l2*, a paralog of the tunneling nanotube-forming M-Sec

**DOI:** 10.1371/journal.pone.0201557

**Published:** 2018-08-07

**Authors:** Paul O’Callaghan, Yvette Zarb, Fredrik Noborn, Johan Kreuger

**Affiliations:** 1 Department of Medical Cell Biology, Uppsala University, Uppsala, Sweden; 2 Department of Neurosurgery, Clinical Neuroscience Center, Zürich University Hospital, Zürich University, Zürich, Switzerland; 3 Department of Clinical Chemistry and Transfusion Medicine, Institute of Biomedicine, Sahlgrenska Academy at the University of Gothenburg, Gothenburg, Sweden; University of Toronto, CANADA

## Abstract

The exocyst is a molecular tether that retains secretory vesicles at the plasma membrane prior to SNARE-mediated docking and fusion. However, individual exocyst complex components (EXOCs) may also function independently of exocyst assembly. Alternative splice variants of EXOC mRNA and paralogs of EXOC genes have been described and several have been attributed functions that may be independent of the exocyst complex. Here we describe a novel splice variant of murine *Exoc3l2*, which we term *Exoc3l2a*. We discuss possible functional implications of the resulting domain excision from this isoform of EXOC3L2 based on structural similarities with its paralog M-Sec (EXOC3L3), which is implicated in tunneling nanotube formation. The identification of this *Exoc3l2* splice variant expands the potential for subunit diversity within the exocyst and for alternative functionality of this component independently of the exocyst.

## Introduction

The exocyst complex is composed of eight distinct components (EXOC1-8) and tethers secretory vesicles to the plasma membrane prior to SNARE-mediated fusion and exocytosis. Isoform variants exist for several of the EXOCs and these are derived from alternative splicing of the canonical EXOC transcripts or paralogous gene expression. We have previously reported on the EXOC3 paralog EXOC3L2 and established that it is required for directional migration of endothelial cells and can associate with other EXOCs [1]. Alternative splicing of *EXOC7* permits isoform switching during epithelial to mesenchymal transition (EMT) in breast cancer cells, such that the mesenchymal EXOC7 isoform promotes a migratory phenotype through its ability to recruit remodelers of the actin cytoskeleton [2]. It remains to be determined whether these alternative EXOC isoforms operate independently of the exocyst; furthermore, if recruited to the exocyst, it is unclear if they replace their canonical EXOC counterparts or can be integrated in addition to them.

M-Sec is another paralog of *Exoc3* and is also known as tumor necrosis factor α-inducible protein 2 (TNFαIP2) and EXOC3L3. M-Sec plays a key role in the formation of tunneling nanotubes (TNTs)[3]. TNTs are fine intercellular membrane connections that are implicated in the transport of organelles and protein complexes [4, 5]. A crystal structure for M-Sec was recently published and highlights its structural similarities with yeast EXOC3 (Sec6) and mouse EXOC7 (Exo70) [6]. Importantly, M-Sec’s ability to induce TNTs relies on its interaction with the GTPase Ral and the exocyst [3]. Studies of the membrane-bending capacity of the exocyst have largely focused on EXOC7, where its ability to dimerize and generate membrane protrusions has lead to its comparison with the inverse BAR (I-BAR) proteins [7], which induce similar membrane deformations and like EXOC7 and EXOC3L2 are required for directional cell migration. Furthermore, EXOCs have also been identified as effectors of the HIV Nef-1 protein, mediating the formation of nanotubes that facilitate intercellular virus transfer [8]. Together these findings indicate that EXOCs and their paralogs serve central membrane-bending functions, including the formation of TNTs.

Here we report an alternative splice variant of murine *Exoc3l2* and using a structural homology model based on M-Sec we compare the properties of the canonical isoform of EXOC3L2 with this alternative isoform. The existence of this splice variant of *Exoc3l2* expands the potential for EXOC3L2 functional diversity, as an integrated component of the exocyst or as an independent agent, and will need to be accounted for in future studies of EXOC3L2.

## Materials and methods

### Ethical permission

C57BL6 adult mice were acquired from Charles River Laboratories and housed at the National Veterinary Institute, Uppsala, Sweden. The animal experiments described in this study were conducted under the ethical permit C222/11, which was granted and approved by the Uppsala Animal Experiments Ethics Board (Uppsala Djurförsöksetiska Nämnd), Uppsala District Court, Uppsala, Sweden.

### RNA isolation and cDNA synthesis

RNA was isolated from mouse tissues using the E.Z.N.A. total RNA isolation kit (Omega Bio-Tek, Norcross, GA, USA) according to the manufacturers protocol for animal tissues homogenized by needle and syringe. The purity and concentration of the RNA isolates were analyzed using a NanoDrop 2000 (NanoDrop, Thermo Fisher Scientific, Uppsala, Sweden). A cDNA library of the RNA isolates was synthesized using the iScript cDNA synthesis kit (BioRad Laboratories, Solna, Sweden) according to the manufacturers instructions.

### Primer design

Primers were designed and confirmed to target the templates of interest using the Primer-BLAST tool hosted at the National Center for Biotechnology Information (NCBI) website. Primers that spanned the novel *Exoc3l2* splice junction were manually designed. Primers were obtained using the Invitrogen Custom DNA Oligos service (Thermo Fisher Scientific). The following primers were used in the study: positive strand primers: P^4:5^, GTCACAGACGTGAAGGCTCA; P^9^, GAAGCTCTGGATGGCATCGT; P^10:11^, GTTTCGGCGGCTGGAGTC; P^sj1^, GGAGAGAATGGCTTATTGGCTTG, and negative strand primers: P^11^, CTCGGAGTGTCCTCCAACTG; P^sj2^, CAAGCCAATAAGCCATTCTCTC.

### Polymerase chain reaction (PCR)

PCR was carried out with AmpliTaq Gold^®^ DNA polymerase with Buffer II and MgCl_2_ (Thermo Fisher Scientific). Taq polymerase and Buffer II were added as per the manufacturers instructions; additionally, one PCR volume contained 0.5 mM MgCl_2_, 5% dimethyl sulfoxide (DMSO), 0.1 μM dNTPs, 0.5 μM forward primer, 0.5 μM reverse primer, and 1.5 μl of sample DNA (1 μg/μl). The PCR was performed on a MiniOpticon PCR System (BioRad Laboratories) or a 2720 Thermal Cycler (Applied Biosystems, Thermo Fisher Scientific) with the following protocol: one cycle of 95°C for 3 min; 39 cycles of 95°C for 30 sec; 60°C for 30 sec; 72°C for 1 min; and one cycle of 72°C for 7 min. PCR products and the GeneRuler 100 bp or 1 kb DNA ladder (Thermo Fisher Scientific) were diluted in DNA Gel Loading Dye (6X; Thermo Fisher Scientific) and separated by electrophoresis on 2% agarose gels, prepared in 40 mM Tris, 20 mM acetate and 1 mM EDTA (TAE buffer) with 0.01% GelRed fluorescent DNA stain (Biotium, Hayward, CA, USA). Gels were scanned using a Gel Do EZ imaging system (BioRad Laboratories) or an Odyssey Fc Imaging System (LI-COR Biosciences, Cambridge, United Kingdom) and images were collected using the ImageLab Software (BioRad Laboratories) or Image Studio^™^ Software (LI-COR Biosciences).

### Sanger sequencing

cDNA from bands of interest was extracted from the agarose gel using a QIAquick Gel Extraction Kit (Qiagen, Hilden, Germany) according to the manufacturers protocol for spin-columns. Extracted cDNA concentrations were determined using a NanoDrop 2000 (NanoDrop, Thermo Fisher Scientific). Sanger sequencing was conducted by the Uppsala Genome Center, Science For Life Laboratory, Uppsala, Sweden. Sequence data was viewed and presented using 4Peaks (A. Griekspoor and Tom Groothuis, Nuclobytes B. V., nucleobytes.com) and Adobe Illustrator software.

### Real-time polymerase chain reaction (RT-PCR)

RT-PCR was carried out using SsoFast Evagreen Supermix (BioRad), according to the manufacturers instructions and performed on an iCycler iQ real-time PCR detection system (BioRad). Melt curves were analyzed using iCycler iQ associated software.

### Sequence alignments and structural homology modeling

Multiple sequence alignment for the *Exoc3l2* Sanger sequencing results was performed using the online version of the MAFFT multiple sequence alignment program [9], and the color-coded nucleotide view was prepared using the associated MSAviewer [10]. Alignments of the N-terminal amino acid sequences of murine EXOC3L2 and M-Sec were performed using the MUSCLE (MUltiple Sequence Comparison by Log Expectation) tool hosted on the European Bioinformatics Institute (EMBL-EBI) website. To construct a structural homology model of murine EXOC3L2 the amino acid sequence was submitted to the SWISS-MODEL server hosted on the ExPASy web server [11, 12]. The top template result returned was for the structure of M-Sec/TNFαIP2 deposited at the Protein Database as 5B86 [6]. The graphical representations of M-Sec and the structural homology model of EXOC3L2 were prepared using PyMol (PyMOL Molecular Graphics System, Version 1.3 Schrödinger, LLC).

## Results

### Detection of an alternative splice variant of murine *Exoc3l2*

**Fig 1:**
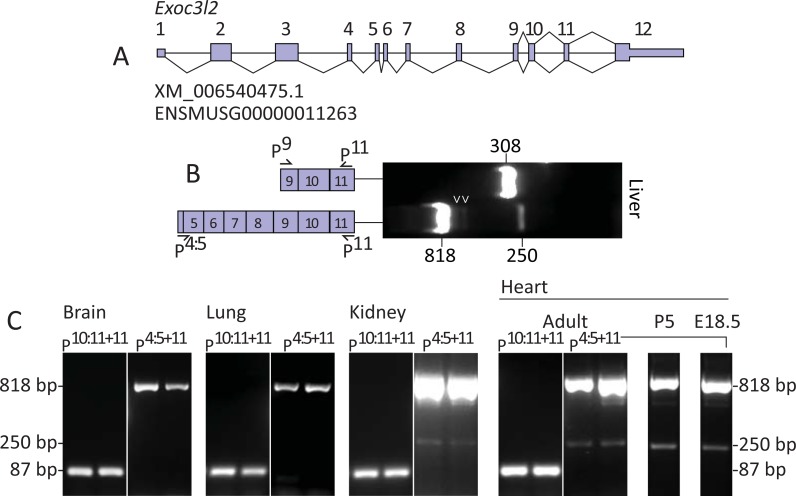
Detection of an alternative splice variant of murine *Exoc3l2*. **A.** Gene map of murine *Exoc3l2* illustrating its twelve exons, as annotated at the NCBI (XM_006540475.1) and Ensembl genome browsers (ENSMUSG00000011263). **B.** PCR of cDNA prepared from RNA isolated from mouse liver using the *Exoc3l2*-specific primers P^9^ and P^11^, and P^4:5^ and P^11.^ **C.** PCR products of cDNA, prepared from RNA isolated from mouse organs, using the EXOC3L2 specific primers P^10:11^ with P^11^, and P^4:5^ with P^11^. Note the second bands detected at 250 bp in kidney and heart tissue in samples amplified with P^4:5+11^.

The murine *Exoc3l2* gene is annotated at the Ensemble database as ENSMUSG00000011263 and at the NCBI database as XM_006540475.1. Two transcript variants of *Exoc3l2* are currently proposed, the longer of the two would encode a 789 amino acid protein (Uniprot D3YUP5) and consists of twelve exons, while the short transcript is derived from the last four exons of the long transcript and would encode a 242 amino acid protein (Uniprot E9Q180; Fig 1A).

**Fig 2:**
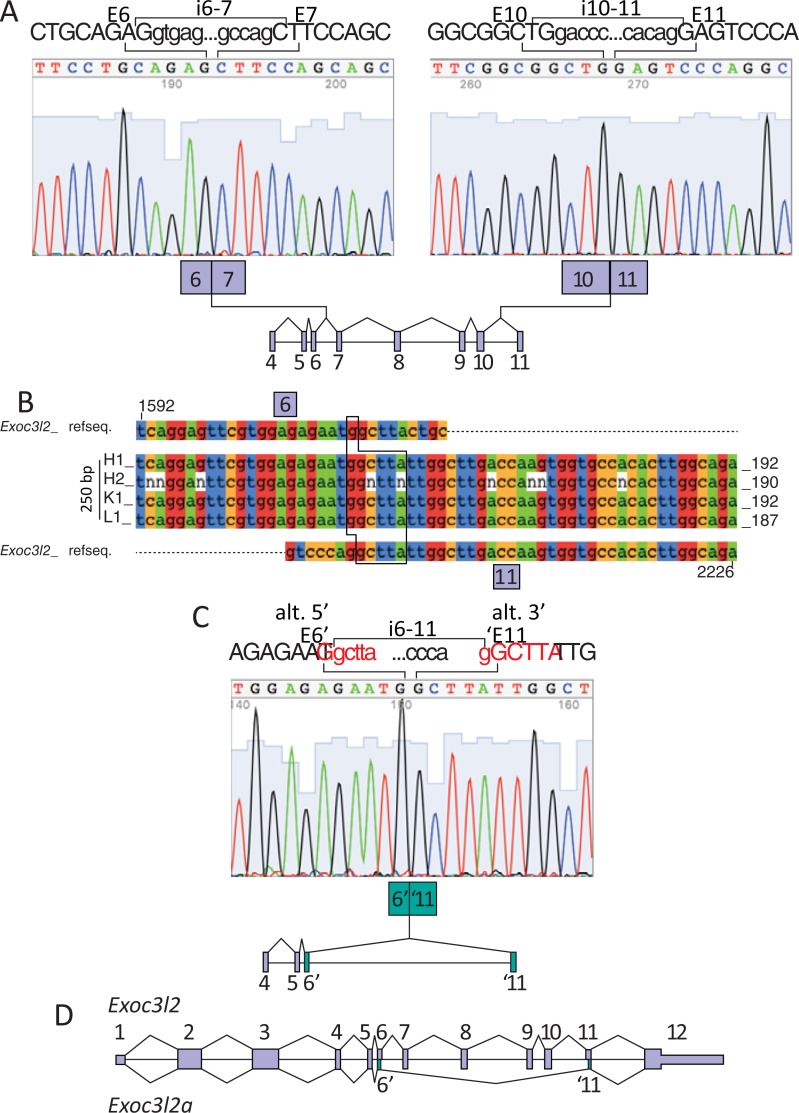
Sanger sequencing of the alternative splice variant of murine *Exoc3l2*. **A.** Sanger sequencing of the 818 bp product confirmed its identity as *Exoc3l2*. The sequence spanning the splice junction between exon 6 (E6) and exon 7 (E7) is presented in the left panel, and that spanning exon 10 (E10) and exon 11 (E11) is presented in the right panel. Above each plot five bases from the intronic sequence that is adjacent to each exons splice site are presented (i.e. i6-7 and i10-11). **B.** Sanger sequencing results from the second 250 bp product amplified using P^4:5^ with P^11^ and cDNA from mouse heart (H1 and H2), kidney (K1) and liver (L1) were aligned using MAFFT software to an *Exoc3l2* mRNA reference sequence. The position of the novel alternative splice site between *Exoc3l2* exon 6 (E6) and exon 11 (E11) was confirmed in each example. The alternatively spliced versions of E6 and E11 are colored in green. **C.** An example of the Sanger sequencing plots for the 250 bp alternatively spliced *Exoc3l2*. The novel splice junction is between an alternative 5’ donor site in exon 6 (E6) and an alternative 3’ acceptor site in exon 11 (E11). Five intronic bases adjacent to E6’ and ‘E11 are presented above the Sanger plot (i.e. i6-11). D. Gene map of murine *Exoc3l2* illustrating the splicing pattern for the canonical (*Exoc3l2*) and alternative (*Exoc3l2a*) variants.

In an effort to detect the long and short transcripts of murine *Exoc3l2* mRNA we designed primers against sites that were specific to the long variants (P^4:5^) or common to both long and short transcripts (P^9^ and P^11^). PCR of mouse liver cDNA with primers P^9^ and P^11^ produced an amplicon that migrated on agarose gel close to the predicted *Exoc3l2* product length of 308 bp, while primers P^4:5^ and P^11^ produced an amplicon that migrated close to the predicted *Exoc3l2* product length of 818 bp (Fig 1B). Unexpectedly, the PCR with P^4:5^ and P^11^ produced a second amplicon of approximately 250 bp, and two additional bands below the 818 bp position were also weakly detected (Fig 1B). The 250 bp product was also amplified and detected in cDNA samples of mouse kidney and various stages of heart development, but not in brain or lungs (Fig 1C). However, it should be noted that the *Exoc3l2* 818 bp band was also considerably weaker in these tissues (Fig 1C and S1 Fig); therefore, the absence of the 250 bp may simply reflect the detection limits of our PCR protocol in tissues with low expression of *Exoc3l2* gene products. To determine whether these unpredicted products were derived from *Exoc3l2* transcripts the bands were excised and subjected to Sanger sequencing using the P^4:5^ primer. As predicted, the 818 bp band was confirmed to be derived from *Exoc3l2* mRNA and examples of the sequencing results across the junctions between exons 6 and 7, and exons 10 and 11 are presented in Fig 2A.

**Fig 3:**
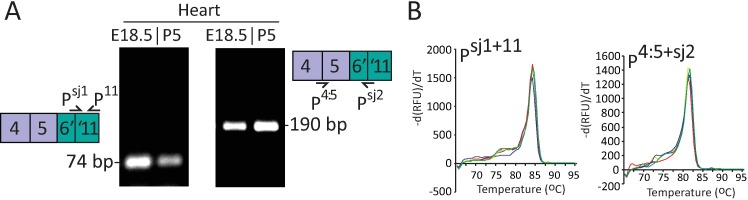
Design of splice site-specific primers for *Exoc3l2a*. **A.** A forward (P^sj1^) and reverse (P^sj2^) primer specific to the alternative splice junction between E6 and E11 of *Exoc3l2* were designed. PCR of cDNA prepared from RNA isolated from mouse heart (E18.5 and P5) using P^sj1^ and P^11^ or P^4:5^ and P^sj2^ yielded single products, of predicted sizes. **B.** Melt curve profiles following RT-PCR of adult mouse heart cDNA (*n* = 5) with P^sj1^ and P^11^ (left plots) or P^4:5^ and P^sj2^ (right plots).

Neither of the bands directly below the 818 bp position (Fig 1B; arrowheads) were identified as *Exoc3l2*, but the 250 bp band was confirmed to be an alternative splice variant of *Exoc3l2*, which we refer to here as *Exoc3l2a*. Sanger results from the 250 bp bands excised from a selection of samples, including mouse heart, kidney and liver all revealed identical sequences for this *Exoc3l2a* variant (Fig 2B). The sequencing results revealed an alternative 5’ splice donor site in exon 6, shortening the exon by 41 bases, and an alternative 3’ splice acceptor site in exon 11, shortening the exon by 9 bases. Exon 6 and exon 11 both contain *GGCTTA* sequences that are reduced to a single *GGCTTA* sequence across the new splice junction (Fig 2C). An established non-consensus splice site motif consists of a *GC* dinucleotide at the 5’ end of an intron and an *AG* dinucleotide at the 3’ end of the intron [13, 14]. We propose that the alternative splice junction for the *Exoc3l2a* transcript connects the first *G* from the *GGCTTA* in exon 6 and the second *G* from the *GGCTTA* in exon 11 as, prior to splicing, these donor and acceptor sites would be separated by an intron consisting of the 5’ *GC-AG* 3’ splice motif (Fig 2C). A gene map illustrating the relative positions of the exon junctions for the canonical and proposed alternative splice variant of *Exoc3l2* is presented in Fig 2D. In an effort to specifically detect the *Exoc3l2a* transcript we designed a forward (P^sj1^) and reverse (P^sj2^) primer that targeted the alternative splice junction between exon 6 and 11 (Fig 3A). PCR of mouse heart (E18.5 and P5) cDNA with P^sj1^ and P^11^ amplified a single product that migrated on agarose gel as a 75 bp band, while P^4:5^ with P^sj2^ amplified a single product that migrated as a 190 bp band, as predicted for these respective fragments of the *Exoc3l2a* transcript (Fig 3A). Analysis of the melt curves following RT-PCR with adult mouse heart cDNA and P^sj1^ with P^11^, or P^4:5^ with P^sj2^ also confirmed that each reaction only generated a single product (Fig 3B).

### Structural assessment of EXOC3L2 modeled on the tunneling nanotube forming M-Sec

**Fig 4:**
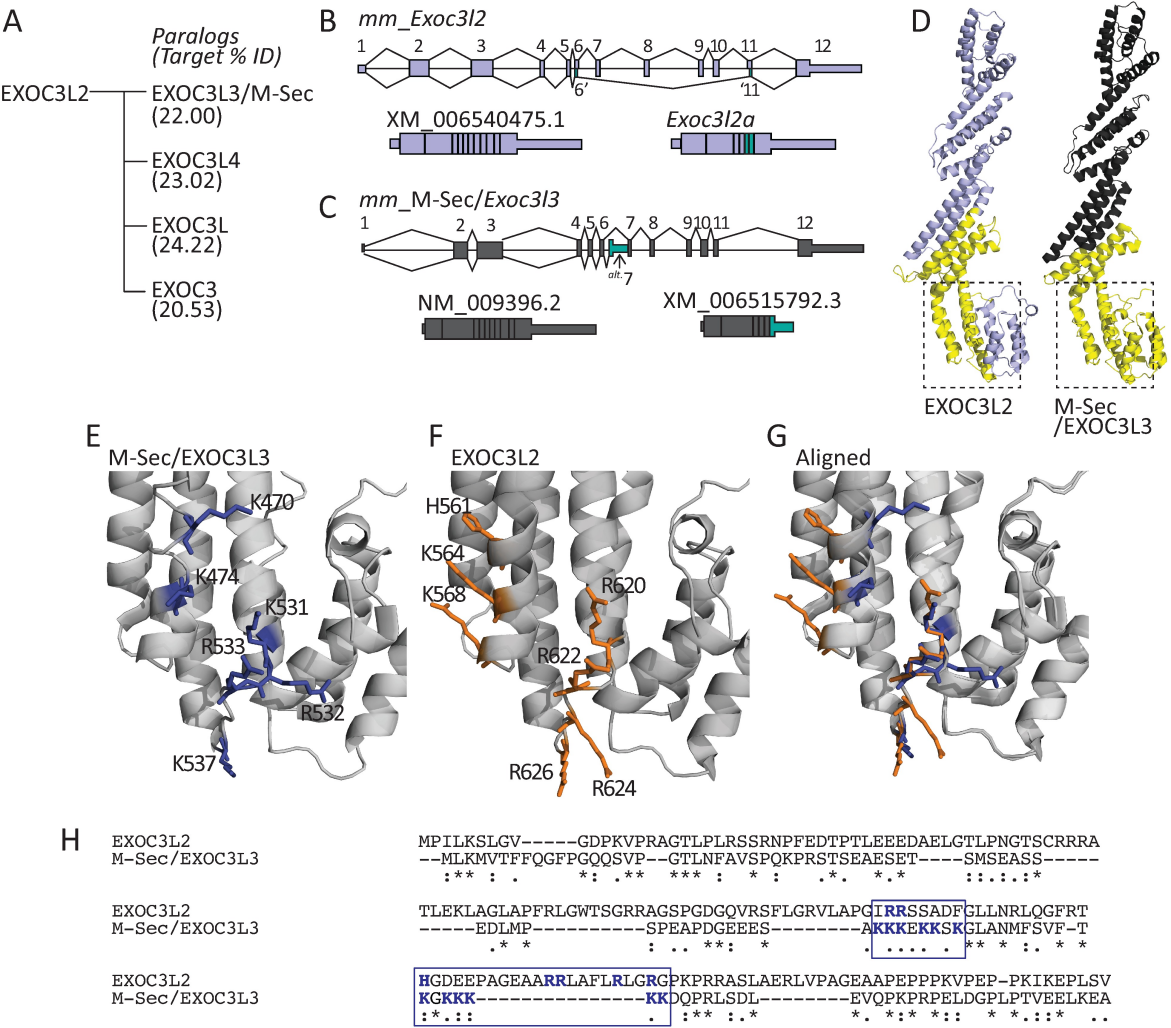
Comparison of EXOC3L2 with the tunneling nanotube-forming M-Sec (EXOC3L3). **A.** Murine *Exoc3l2* has four paralogs encoding the proteins EXOC3L3 (aliases: TNFαIP2/M-Sec), EXOC3L4, EXOC3L and EXOC3. The % amino acid identity between the protein derived from each paralog and EXOC3L2 is presented in brackets. **B.** Gene map of murine *Exoc3l2*, a schematic view of the *Exoc3l2* and *Exoc3l2a* mRNA transcripts are presented beneath the map. **C.** Gene map of murine M-Sec/*Tnf*α*ip2*/*Exoc3l3* illustrating the splicing pattern for the canonical (NM_009396.2) and a potential alternative splice variant (XM_006515792.3). **D.** Structural homology model of EXOC3L2 generated using SWISS-MODEL and based on the structure of murine M-Sec (PDB: 5B86)., the yellow domain would be excised from EXOC3L2a (left structure). Model of murine M-Sec/EXOC3L3, the yellow domain would be lost from the proposed slice variant of M-Sec (right structure). **E.** Two regions in the C-terminus of M-Sec that are functionally important for the formation of TNTs are illustrated as blue sticks on the grey helical backbone. **F.** EXOC3L2 amino acids with similar properties to those found in M-Sec’s region 1 and 2 are illustrated as orange sticks on the grey helical backbone. **G.** Alignment of the EXOC3L2 and M-Sec models. The regions illustrated in **E** and **F** are those framed with dotted lines in **C** and **D**, respectively. **H.** MUSCLE sequence alignment of EXOC3L2 with M-Sec. The two polybasic patches in the N-terminus of M-Sec and the aligned sequence of EXOC3L2 are framed in blue, and the positively charged amino acids in these regions are in bold blue text.

In an effort to visualize the impact that this alternative splicing would have on the EXOC3L2 protein we generated a structural homology model using the SWISS-MODEL server. The crystal structure of TNFαIP2, deposited with identifier 5B86 at the Protein Database, was returned as the best template match. TNFαIP2 is also known as M-Sec and EXOC3L3, and its gene is one of four murine paralogs of *Exoc3l2*, the others being *Exoc3*, *Exoc3l* and *Exoc3l4* (Fig 4A). The M-Sec protein shares 22% amino acid identity with the 789 amino acid isoform of EXOC3L2 (Fig 4A). The M-Sec gene is annotated as NM_009396.2 at the NCBI database, with an additional splice variant recorded as XM_0065157922.3 (Fig 4C). The predicted alternative site for M-Sec utilizes an alternative exon 7, which encodes an early stop codon, leading to a truncated protein lacking the residues encoded by exons 8–12. While not identical to the alternative splicing of *Exoc3l2*, there is a large degree of overlap between the transcript region excised from the proposed M-Sec splice variant and *Exoc3l2a* (Fig 4B and 4C). The regions in both proteins that would be missing from the isoforms translated from their respective splice variant transcripts are illustrated in Fig 4D. Two regions consisting of K470 and K474; and K531, R532, R533 and K537 in the C-terminus of M-Sec have been implicated as essential for the proteins ability to promote the formation of TNTs (Fig 4E) [6]. The homology model of EXOC3L2 reveals that these regions are also populated by basic amino acids in similar positions on the respective α-helices; namely H561, K564 and K568, and R620, R622, R624 and R626 (Fig 4F). Aligning the M-Sec and EXOC3L2 structures reveals that the best overlap is observed in the positioning of the residues in the second patch of basic amino acids (Fig 4G). Two additional polybasic patches (KKEKKSK and KGKKKKK) in the N-terminus of M-Sec are important for locating the protein to the plasma membrane [6]. This region of M-Sec is not part of the 5B86 structure, but a MUSCLE sequence alignment of EXOC3L2 and M-Sec indicates that EXOC3L2 contains two basic amino acids in the region that aligns to the first M-Sec polybasic site, and five basic amino acids in the second site (Fig 4H).

## Discussion

Here we define a novel splice variant of murine *Exoc3l2*. Several EXOCs are known to undergo alternative splicing and two isoforms of EXOC7 have opposing effects on the invasiveness of cancer cells during EMT [2]. Therefore, identifying EXOC splice variants can prove central to revealing their diverse functional repertoire. The novel splice site identified here for *Exoc3l2a* represents a non-consensus splice motif, which consists of a *GC-AG* intron. Thanaraj et al. report that for such motifs it is common that both the donor and acceptor sites are alternative [14], as demonstrated here for *Exoc3l2a*.

The recently published structure of M-Sec reveals similarities with a number of tethering factors including Sec6 the yeast ortholog of EXOC3 [6]. Kimura et al. conclude that the polybasic patches in M-sec’s N terminus associate it to the plasma membrane, while key basic residues in its C terminus interact with RalA and in doing so recruit the exocyst complex [6]. The importance of RalA and the exocyst complex has also been established in the context of cytokine-induced filopodia formation [15], suggesting a general role for the exocyst in the formation of membrane protrusions. The structural homology model of EXOC3L2 presented here indicates that it may also possess RalA binding properties similar to M-Sec. Importantly, these potential RalA binding domains would be missing from both the alternatively spliced M-Sec isoform XM_0065157922.3 and EXOC3L2a. The functional relevance of these shortened variants of the canonical proteins is difficult to predict, but they would presumably be unable to participate in recruitment of the exocyst complex, which in the case of M-Sec is essential for TNT formation. To date EXOC2 (Sec5) and EXOC8 (Exo84) are the only EXOCs that have been established as having RalA binding domains [16, 17]; therefore, the capacity of EXOC3L2 to bind RalA has yet to be established. However, EXOC3L2 does bind to EXOC4, indicating that its role in directed endothelial cell migration is likely fulfilled as an integrated component of the exocyst [1]. It will therefore be relevant to determine if EXOC3L2a can support this function or if critical binding domains are lost due to the splicing event.

While a shared evolutionary origin explains the structural similarities between tethering proteins and their paralogs [18], the functional relevance of these similarities has yet to be fully elucidated. The rod-like structure of EXOCs has been suggested to facilitate subunit packing during exocyst assembly [19]. Therefore, it will be relevant to determine whether EXOC3 paralogs, particularly M-Sec and EXOC3L2, are interchangeable subunits that are selectively assembled into different functional variants of the exocyst complex, or independent operators.

EXOC7 is the exocyst component with an established capacity to induce membrane protrusions and recruit actin remodelers [2, 7]. These functions have primarily been studied in the context of cell migration, but are clearly applicable during the formation of TNTs, which have been demonstrated to contain cytoskeletal structures [5, 20]. The C terminal domain of Sec6 (yeast EXOC3) is sufficient to bind Exo70 (yeast EXOC7)[21]; therefore, it is conceivable that EXOC3 and its paralogs are capable of recruiting EXOC7 to sites at which membrane remodeling is required. Given that a large region of the C-terminal domain is lacking in EXOC3L2a, this splice variant is unlikely to support such interactions with EXOC7.

Notably, Tress *et al*. discuss that while transcript analysis suggests a prevalence of alternative splicing events, the ever increasing body of proteomic datasets suggests that there are relatively few alternative isoforms expressed [22]. Such studies generate highly informative datasets, but are often limited by the number of contexts in which samples can be assessed. As discussed above, EXOC7 undergoes isoform switching during EMT in cancer cells, adopting functions that are beneficial to disease progression [2]. Therefore, the expression of at least some alternatively spliced transcripts may prove to be context-dependent.

It remains to be confirmed whether the *Exoc3l2a* transcript is translated and if so what potential functional implications are incurred through the loss of the excised sequence. Alternative splicing can also function as a transcriptional regulator, such that alternatively spliced mRNA is targeted for decay due to the inclusion of ‘poison exons’ [23]. In this respect, transcription of *Exoc3l2a* may occur at the cost of *Exoc3l2* and thus lead to a suppression of EXOC3L2 expression. However, the alternative splice sites identified for *Exoc3l2a* simply exclude part of the mRNA sequence of *Exoc3l2* and the reading frame remains intact after the alternative splice junction. Therefore, there is no apparent reason why the *Exoc3l2a* transcript would be targeted for nonsense mediated decay and it seems reasonable to assume that it is available for translation.

A polymorphism (rs597668) near the *EXOC3L2* gene locus has been associated with Alzheimer’s disease (AD) [24], and while no role for EXOC3L2 in AD has yet been proposed it may prove relevant to consider the expression and functionality of alternatively spliced EXOC3L2 isoforms in the context of AD. From a practical standpoint, knowledge of this alternatively spliced variant of *Exoc3l2* is important when designing primers and probes for studying transcript expression, and also when attempting to target specific epitopes of the translated protein with antibody based techniques.

## Conclusions

Using a strategy of reverse transcription PCR, exon junction-specific primers and Sanger sequencing we have identified a novel splice variant of murine *Exoc3l2*, and confirmed its expression in a number of major organs. Based on the recently published structure of M-Sec we built a structural homology model of EXOC3L2 and observed that the C-terminal region lost from this alternatively spliced isoform is similar to that lost from a proposed splice variant of M-Sec (XM_006515792.3). This region contains key residues implicated in M-Sec’s ability to recruit RalA and the exocyst and in doing so facilitate TNT formation. Residues with similar properties are found at comparable locations within the region lost from this alternatively spliced isoform of EXOC3L2. Identification of this splice variant of murine *Exoc3l2* expands the family of exocyst-like proteins and future studies are warranted to determine the functional relevance of this alternatively spliced isoform of EXOC3L2.

## Supporting information

S1 Fig Relative *Exoc3l2* transcript expression in mouse heart (adult) and brain. Equal concentrations of mouse heart (adult) and brain cDNA were amplified using primers P4:5+11 and P10:11+11 for Exoc3l2 and the β-actin house keeping gene ACTB. The similar strength of ACTB bands in heart and brain samples indicates that equal concentrations of cDNA (determined prior to PCR by nanodrop) were used from both tissues; therefore, the relatively weaker 818 bp bands for Exoc3l2 in brain samples suggests lower levels of Exoc3l2 transcription in this tissue. Bands at 250 bp representing the splice variant Exoc3l2a were detected in 2/3 mouse heart samples, but in none of the three brain samples.
(EPS)
